# The Requisite Conditions for Practicing Dentistry in the Province of Quebec

**Published:** 1888-06

**Authors:** 


### Editorial, Etc.

---

THE REQUISITE CONDITIONS FOR PRACTICING DENTISTRY IN THE PROVINCE OF QUEBEC.—Before entering upon the study of dentistry in the province of Quebec, every person must, previous to signing indentures with a licentiate, present to the Secretary of the Board a certificate of having satisfactorily passed the matriculation examination prescribed by law. The Canadian practitioners are naturally proud of the matriculation examination which is required before the period of studentship can count, and which is very similar to our own, both as regards obligatory and optional objects. The period of studentship comprises four years of actual service in the office of a licentiate, and the attendance after the second year upon at least one full course of lectures in a recognized dental or medical college upon the following subjects:—Anatomy (theoretical and practical), Physiology and Chemistry. In practical anatomy, the student must give proof of having dissected at least one head and neck. Certified tickets of regular attendance upon each subject are positively required, If a student desires to attend a Dental College, the actual time of such attendance is accepted as an equivalent to the same period of studentship. Students in their third and fourth years are supplied with a brief synopsis of studies, comprising an outline of the special subjects of examination.

After the usual requirements as to age, matriculation and studentship, the candidate is required to lodge with the treasurer a fee of sixty dollars, forty of which are refunded him in case of failure. A preliminary practical examination takes place, in which the student is required to perform operations in the mouth, and to give practical evidence of his skill as a mechanical dentist. The examination is written, oral and clinical, and divided as follows:—

1. Dental Anatomy and Physiology (Head and Neck).
2. Chemistry and Metallurgy.
3. Anæsthetics, Dental Hygiene.
4. Operative Dentistry (Theoretical and Practical).
5. Mechanical Dentistry " "
6. Dental Pathology, Therapeutics and Materia Medica.
7. Irregularties of the Teeth (Causes and Treatment).
8. Origin and Development of the Teeth.

Since 1885, every applicant has also been obliged to lodge with the secretary an original thesis upon some practical subject in dentistry, which he must defend before the board of examiners.

Since 1884, the annual assessment of two dollars has been imposed upon each licentiate, the only penalty for non-payment, however, seems to be that no licentiate in arrears is entitled to vote at the election of the Board of Examiners.

Another interesting resolution was passed in the same year, by which licentiates are not allowed to open branch offices under the charge of students or other unlicensed parties, and such students or other unlicensed parties are subject to be prosecuted if detected.

Provided the usual statutory notice of intended amendment or addition is duly made, any section of the by-laws, or any of the rules of order or regulations of the Board may be amended or additions made by a three-fifths vote of the members present.

The election of the Board of Examiners, seven in number is made by ballot every three years, at the usual general meeting. This Board also conducts the general management of the affairs of the Association The members of the Board are entitled to a fee not exceeding five dollars per diem, in addition to their traveling expenses, for every day that the Board shall sit. The Board has also power, upon a complaint made by any person lawfully practising the profession of dentist, to bring before the Board any member of the said Association accused of infringing the by-laws passed by the Board or of any act derogatory to the honor or dignity of the profession of dentist, or of exercising a calling, trade or industry incompatible with such profession. The secretary is empowered to administer the oath or affirmation to witnesses on behalf of the complainant or of the accused, and also to summon before the Board any person who may be called upon to give evidence in the manner prescribed in the Code of Civil Procedure. Any person refusing to comply with this summons incurs a penalty not exceeding twenty dollars for each infringement. The Board has the power to censure and reprimand any member found guilty of any of the above enumerated offences, prevent him from attending and taking part in the meetings of the members of the said Association for a period not exceeding three years, and may even, according to the gravity of the offence, suspend the said member from the exercise of his profession in the province of Quebec for a period not exceeding one year. Every accused member who considers himself aggrieved by any decision of the Board may appeal to a general meeting of the Association, provided the appellant deposits the sum of one hundred dollars as a security for payment of the costs occasioned by the calling of the general meeting and of those of the complainant. Every dentist found guilty of unlawful practices or of felony, or deprived by a civil interdiction of his civil rights, *ipso facto* ceases to have the right to practice as a dentist in the province and the Board must delete his name from the list of members. The Board may, however; on the application of the person so struck off, reinstate him upon what are deemed proper conditions to impose.

"After the passing of this Act every person who, not being the bearer of a dentist's license granted by the Board of Examiners, constituted in virtue of the Act 37 Vict., chap. 14, shall practice in this province the profession of dentist for a remuneration or in hope of a reward or of payment, either directly or indirectly, or who attempts to elude the law by causing his services as dentist to be indirectly paid by means of the sale of drugs or medicines, or who willingly and falsely claims to be in the posession of a license granted under this Act, or the Acts above mentioned, or shall falsely make use of names, titles and qualities calculated to convey the impression that he is authorised to practice the profession of dentist, or shall make use of any title calculated to convey the impression that he is a graduate of a college of dentists, may, upon complaint or information sworn to before a justice of the peace, made by a person of at least twenty-one years of age, be brought by a simple writ of summons or by a warrant of arrest in the discretion of any court of competent jurisdiction, and, upon conviction of any of the offences above mentioned, be condemned to pay a fine not exceeding two hundred dollars, with costs, for each offence, and in default of payment of such fine and costs, the defendant may be condemned to an imprisonment in the common gaol of the district in which the sentence has been pronounced for a period not exceeding three months, unless the said penalty and costs be sooner paid.

"If the person so found guily of any of the offenses above enumerated should be again accused and convicted of any of the offences above enumerated, he shall then be condemned to pay a fine not exceeding four hundred dollars including costs, for each offense, and, in default of payment, to an imprisonment not exceeding six months, unless the said penalty and costs be sooner paid.

"No person so unlawfully practicing the profession of dentist shall recover before any court of justice any sum of money for the professional services so rendered, or for the medicines or other materials supplied in such unlawful practice.

"The fines imposed by this Act shall be payable to the treasurer of the Associstion, and shall form part of the funds of such Association.

"Nothing in this act shall be construed as restricting the privileges conferred upon physicians and surgeons in the province of Quebec by the various Acts relating to the practice of medicine and surgery therein . . . . . but in the event of a physician or surgeon desiring to practice as a dentist and to be publicly known as such, he shall before doing so be obliged to obtain a license from the Board of Examiners of such Association, by submitting to an examination upon the mechanical and practical part of dental surgery and by paying the fee fixed by the bye-laws for the obtaining of such license."

The number of dentists practicing in the province of Quebec only amount to sixty.—*Dr. Geo. Cunningham in a letter to London Dental Record.*

